# Swimming training prevents coronary endothelial dysfunction in ovariectomized spontaneously hypertensive rats

**DOI:** 10.1590/1414-431X20165495

**Published:** 2017-01-16

**Authors:** E.R.G. Claudio, S.A. Almeida, V. Mengal, G.A. Brasil, C.H. Santuzzi, R.V. Tiradentes, S.A. Gouvea, N.S. Bissoli, R.L. Santos, G.R. Abreu

**Affiliations:** Departamento de Ciências Fisiológicas, Centro de Ciências da Saúde, Universidade Federal do Espírito Santo, Vitória, ES, Brasil

**Keywords:** Swimming training, Ovariectomy, Coronary reactivity, Oxidative stress, Antioxidant enzymes

## Abstract

Estrogen deficiency and hypertension are considered major risk factors for the development of coronary heart disease. On the other hand, exercise training is considered an effective form to prevent and treat cardiovascular diseases. However, the effects of swimming training (SW) on coronary vascular reactivity in female ovariectomized hypertensive rats are not known. We aimed to evaluate the effects of SW on endothelium-dependent coronary vasodilation in ovariectomized hypertensive rats. Three-month old spontaneously hypertensive rats (SHR, n=50) were divided into four groups: sham (SH), sham plus swimming training (SSW), ovariectomized (OVX), and ovariectomized plus swimming training (OSW). The SW protocol (5 times/week, 60 min/day) was conducted for 8 weeks. The vasodilatory response was measured in isolated hearts in the absence and presence of a nitric oxide synthase inhibitor (L-NAME, 100 µM). Cardiac oxidative stress was evaluated *in situ* by dihydroethidium fluorescence, while the expression of antioxidant enzymes (SOD-2 and catalase) and their activities were assessed by western blotting and spectrophotometry, respectively. Vasodilation in SHR was significantly reduced by OVX, even in the presence of L-NAME, in conjunction with an increased oxidative stress. These effects were prevented by SW, and were associated with a decrease in oxidative stress. Superoxide dismutase 2 (SOD-2) and catalase expression increased only in the OSW group. However, no significant difference was found in the activity of these enzymes. In conclusion, SW prevented the endothelial dysfunction in the coronary bed of ovariectomized SHR associated with an increase in the expression of antioxidant enzymes, and therefore may prevent coronary heart disease in hypertensive postmenopausal women.

## Introduction

Coronary heart disease (CHD) is the main source of morbidity and mortality in the world (1), for which hypertension is considered a great risk factor (2). Hypertension causes a significant increase in the coronary vascular resistance, impairs the auto-regulatory mechanism and can cause the left ventricle (LV) dysfunction (3).

Women in their reproductive period are protected against cardiovascular diseases (CVD), mainly because of estrogens. However, the decline in estrogens production in the postmenopausal period can raise the risk for the development of hypertension and, consequently, CHD (4,5). Animal studies showed that ovariectomy increases blood pressure (BP) of normotensive rats (6) and even worsens the hypertensive state of spontaneously hypertensive rats (SHR) (7,8).

Oxidative stress is a common feature of hypertension and of estrogen deficiency (6,9,10), and helps to promote endothelial dysfunction (11). This pathological modification in endothelial biology is of great importance because the impairment of endothelium vasodilation in human coronary circulation predicts future cardiovascular events and long-term outcomes (12).

On the other hand, exercise training (ET) has been considered an effective method to prevent or treat many CVDs. Accordingly, ET can lower BP in humans (13) and in male SHR (14), but its effects are controversial in female SHR (15,16). The anti-hypertensive effects of ET have been attributed mainly to neurohumoral and structural adaptations, increased vascular responsiveness to vasodilator stimuli, or both. These responses include an increase in the plasmatic atrial natriuretic peptide concentration (14), enhancement of nitric oxide (NO) production and a decrease in angiotensin II levels (17), attenuation of arterial stiffness in ovariectomized (OVX) rats (18), and a reduction in oxidative stress (19).

In addition, engaging in daily ET has been shown to improve reactive hyperemia and endothelium-dependent dilation in normotensive and hypertensive human subjects by an augmented NO formation (13,20).

Nevertheless, although ET may cause a reduction in BP in hypertensive animals, the relationship between ET and the endothelium-mediated coronary reactivity in female ovariectomized SHR is not known. This is of major importance given the potential additive effects of high BP and estrogen deficiency on the impairment of coronary circulation that may be observed in postmenopausal hypertensive women. Our hypothesis was that swimming training (SW) may prevent the endothelial dysfunction in the coronary circulation of ovariectomized SHR. Thus, the aim of this study was to evaluate the effects of 8 weeks of SW on the endothelium-mediated dilation in the coronary bed in a model of postmenopausal hypertension. Furthermore, we sought to evaluate the oxidative stress and the enzymatic antioxidant system in order to verify the adaptive responses to ET on the reactive oxygen species production.

## Material and Methods

### Animals

Three-month-old female spontaneously hypertensive rats (n=50) were obtained from the university facility. All procedures were approved by the Institutional Ethical Committee for Animal Care and Use of the Universidade Federal do Espírito Santo, Vitória, ES, Brazil (protocol #024/2011). Experiments were conducted in accordance with the Guide for the Care and Use of Laboratory Animals published by the US National Institutes of Health (NIH Publication, revised 1996). Animals were kept in communal cages with free access to water and standard rat chow (Purina Labina¯, Brazil). Temperature (22-24°C), humidity (40-60%), and light-dark cycles (12-12 h) were carefully controlled. At the time of ovariectomy, the animals were randomly divided into four groups as follows: sham (SH), sham plus swimming training (SSW), ovariectomized (OVX), ovariectomized plus swimming training (OSW).

### Ovariectomy

Ovariectomy was performed under anesthesia with ketamine (80 mg/kg, *ip*) and xylazine (12 mg/kg, *ip*). Bilateral dorsolateral incisions were made through the skin, and the underlying muscle tissue was dissected to locate the ovaries and fallopian tubes, which were ligated with a suture line, and the ovaries were removed. The muscle and skin were then sutured with an absorbable suture. After surgery, animals received an injection of antibiotic (2.5% enrofloxacin, 0.1 mL, *im*). In sham animals, the same procedure was followed but without ovariectomy. The surgery was done in all animals during the same period of the day and seven days of recovery were allowed before the start of the training protocol.

### Swimming training

The swimming training was performed according to the protocol described by Kuru et al. (21), in an apparatus adapted for rats. It contained warm water (30-32°C) and had a depth of 60 cm. The training protocol was conducted during the same period of the day (4:00-6:00 pm) for all the training sessions. The first week consisted of an adaptation period, initiated with 10 min of continuous swimming training on the first day. Swimming time was increased daily until reaching 60 min at the end of the fifth day. From the second week, the exercise duration was kept constant (60 min/day, 5 days/week) with 2 days of rest. This was maintained until the end of the training period, which lasted 8 weeks. To avoid effects related to acute exercise, animals rested for 48 h before being sacrificed for all additional procedures (22).

### BP measurements

Systolic BP (SBP) was evaluated in conscious rats before and after the training period and was determined by an indirect tail-cuff method (IITC Life Science, Inc., USA). Animals were restrained for 5-10 min and conditioned to the procedure with cuff inflation-deflation cycles. The results of three stable measurements of SBP were averaged. The pressure was automatically controlled and systolic pulses were detected by a pulse transducer. A suitable cuff size was selected for each animal.

### Isolated heart preparation (modified Langendorff method)

To evaluate coronary perfusion pressure (CPP) and endothelium-dependent vasodilation, animals were anesthetized with chloral hydrate (40 mg/kg, *ip*). The rats were sacrificed by decapitation, and their hearts were immediately excised and perfused at a constant flow. Studies on the coronary vascular bed were performed on whole hearts using a Langendorff preparation (Hugo Sachs Electronics, Germany), for perfused isolated hearts (23,24). Briefly, the isolated hearts were perfused with modified Krebs solution containing 120 mM NaCl, 1.26 mM CaCl_2_·2H_2_O, 5.4 mM KCl, 2.5 mM MgSO_4_·7H_2_O, 2 mM NaH_2_PO_4_·H_2_O, 27 mM NaHCO_3_, 1.2 mM Na_2_SO_4_, 30 μM EDTA, and 11 mM glucose. The solution was equilibrated with 95% oxygen and 5% carbon dioxide at a controlled pressure of 100 mmHg to give a pH of 7.4. Langendorff preparations were perfused at a rate of 10 mL/min with a peristaltic pump (MS-Reglo 4 channels; Hugo Sachs Electronics) and maintained at a temperature of 37°C. A fluid-filled balloon was introduced into the LV and connected to a transducer (Incor, Brazil) to measure the isovolumetric cardiac force and intrinsic heart rate. The balloon was filled with a spindle syringe until it reached a pre-load of 10 mmHg. The baseline CPP was measured after a period of stabilization (40 min). Endothelium-dependent vasodilation was randomly analyzed by an *in bolus* administration of 0.1 mL of bradykinin (Sigma, USA) in concentrations varying between 10^-10^ to 10^-6^ M. To analyze the role of NO in the endothelium-mediated relaxation of sedentary and ovariectomized swimming-trained SHR, we perfused the isolated hearts with the non-selective nitric oxide synthase inhibitor L-NAME (100 µM) for 20 min and then determined the concentration-response curve with bradykinin.

### Isolation of coronary arteries

The thorax cavity was opened and the heart removed and placed in cold Krebs-Henseleit buffer containing 115 mM NaCl, 25 mM NaHCO_3_, 4.7 mM KCl, 1.2 mM MgSO_4_·7H_2_O, 2.5 mM CaCl_2_, 1.2 mM KH_2_PO_4_, 11 mM glucose, and 0.01 mM Na_2_EDTA at pH 7.4, during the dissection procedure. The left anterior descending branch and the septal branch of the left coronary artery were isolated under a dissecting microscope (D.F. Vasconcelos M900, Brazil), freed of surrounding ventricular muscle tissue and snap frozen in liquid nitrogen. The samples were stored at -80°C until their use.

### Detection of superoxide production (dihydroethidium fluorescence)

Dihydroethidium (DHE) fluorescence was used to detect the superoxide production in cardiac tissue as previously described (25). We perform this in the cardiac tissue because the coronary circulation is regulated mainly under the influence of the cardiac metabolism products, including the reactive oxygen species (26). Briefly, frozen, unfixed cardiac sections were cut into 8-mm thick sections and mounted on gelatin-coated glass slides. Then, the samples were incubated with the oxidative fluorescent dye DHE (2 mM) in a modified Krebs’ solution (containing 20 mM HEPES), in a light-protected humidified chamber at 37°C for 30 min, to detect superoxide. The fluorescence intensity was detected at 585 nm and quantified using a confocal fluorescence microscope (Leica DM 2500 TI, Nikon Instruments Inc., USA) by a technician completely blind to the experimental protocol. Analysis of 10 fields per sample was performed.

### Western blotting

Coronary samples were homogenized in a lysis buffer containing 150 mM NaCl, 50 mM Tris-HCl, 5 mM Na_2_EDTA, and 1 mM MgCl_2_, plus a protease inhibitor (Sigma Fast; Sigma). Protein concentration was determined by the Lowry method (27) and bovine serum albumin (BSA) was used as the standard. The same amount of proteins (50 µg) were denatured and separated by SDS-PAGE (10%) and transferred onto a PVDF membrane (Millipore, Germany). Membranes were blocked with 5% BSA at room temperature in a TBS buffer plus Tween 20 (0.1%) before incubation with polyclonal anti-mouse antibody for Mn-superoxide dismutase 2 (SOD-2; 1:1000-Sigma), monoclonal anti-mouse antibody for catalase (1:2000-Sigma), and polyclonal anti-mouse antibody for β-actin (1:1500-Sigma). After washing, the membranes were incubated with an alkaline phosphatase conjugated anti-mouse IgG (1:3000, Abcam Inc., USA). The bands were visualized using an NBT/BCIP system (Invitrogen Corporation, USA) and quantified using ImageJ software (National Institute of Health, USA).

### Antioxidant enzyme activity

SOD activity in cardiac tissue was assessed according to the Misra and Fridovich method (28). Briefly, 0.1 mL of homogenate (0.25 mg/mL) was added to a quartz cuvette containing 1.0 mL of carbonate buffer (0.2 M, pH 10.2), 0.8 mL of KCl (0.015 M). Total volume was completed to 3.0 mL with deionized water. The reaction was initiated by the addition of epinephrine (0.025 M) and the inhibition of autocatalytic adrenochrome formation rate was measured. Measurements were made spectrophotometrically at 480 nm. The enzymatic activity is reported as units of SOD/mg protein. Catalase enzyme activity was measured in the supernatants as described by Nelson and Kiesow (29). Briefly, 0.04 mL of H_2_O_2_ was added as a substrate to 0.06 mL of homogenate and 1.9 mL of potassium phosphate buffer (50 mM, pH 7.0) to give a final H_2_O_2_ concentration of 6 mM. The reaction proceeded for 1 min at room temperature. Decomposition of H_2_O_2_ by catalase was determined by the variation in absorbance at 240 nm (ΔE). Measurements were performed in duplicate. The enzymatic activity is reported as millimoles of H_2_O_2_ decomposed per minute per milligram of protein (ΔE·min^-1^·mg protein^-1^).

### Citrate synthase activity

Citrate synthase activity was measured as previously described (25). Briefly, samples of soleus muscle were homogenized in phosphate buffer (50 mM sodium phosphate, 1 mM EDTA and protease inhibitor cocktail (Sigma-Aldrich, USA), pH 7.4, centrifuged for 15 min at 12,000 *g* and 4°C and the pellet was then discarded. The supernatant was used for the assay. The assay mixture contained 100 mM Tris, 1 mM EDTA, 0.2 mM DTNB, 0.1 mM Acetyl-CoA, 1% (v/v) Triton X-100, sample (130 mg of soluble proteins per mL of total assay) and 0.5 mM oxaloacetate (added last). The absorbance was monitored at 412 nm in a 96-well plate for 10 min at 25°C, and maximal citrate synthase activity was measured within the linear range of the assay.

### Statistical analysis

Data are reported as means±SE. A paired Student’s *t*-test and one- and two-way ANOVA were used, when appropriate. The differences among groups found by ANOVA were tested with the Fisher's *post hoc* tests for multiple comparisons. Statistical significance was set at P<0.05.

## Results

### Body weight, estrogen status, cardiac weight and citrate synthase activity

Body weight was significantly increased by ovariectomy and was not altered by ET in female SHR. Uterus weight (UW) and the ratio of UW to body weight (BW) were used to evaluate the estrogen status. As expected, there was a significant decrease in both parameters in OVX animals (P<0.05). Heart weight (HW) and LV weight (LVW) were not significantly altered by ovariectomy or by swimming training, even when adjusted by body weight. In order to analyze the effectiveness of our swimming training protocol, we measured the citrate synthase enzyme activity of soleus muscle samples. The results showed a significant increase in the enzymatic activity of both trained groups compared to the sedentary groups (P<0.05, Table 1).

**Table:**
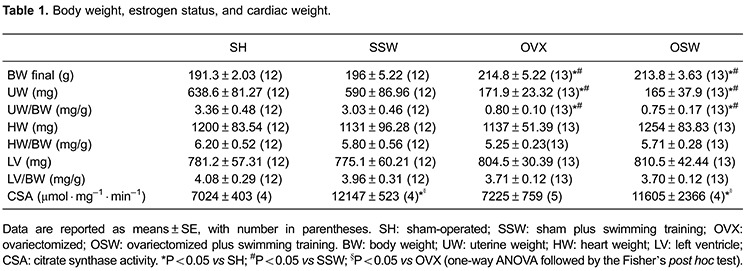

### Systolic BP, baseline CPP and vasodilatory response to bradykinin

Systolic BP was not different between the groups at baseline. However, when the initial and final SBP within each group was compared, we observed a significant increase in the final SBP only in the OVX group (P<0.05), suggesting that ET is able to prevent the worsening of hypertension, which occurs with estrogen deficiency.

**Figure 1:**
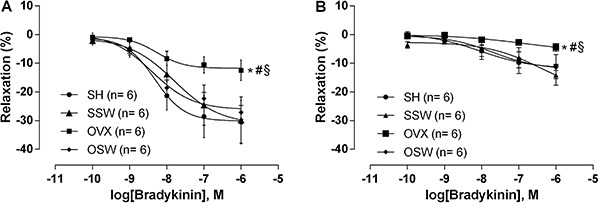
Endothelium-dependent vasodilator response to bradykinin. *A*, Estrogen deficiency in spontaneously hypertensive rats led to endothelial dysfunction, which was prevented by 8 weeks of swimming training. *B*, Pre-incubation with nitric oxide synthase inhibitor (L-NAME, 100 µM) reduced the endothelium-dependent vasodilation in all groups. SH: sham-operated; SSW: sham plus swimming training; OVX: ovariectomized; OSW: ovariectomized plus swimming training. Data are reported as means±SE. *P<0.05 *vs* SH, ^#^P<0.05 *vs* SSW, and ^§^P<0.05 *vs* OSW (two-way ANOVA followed by Fisher's *post hoc* test).

The baseline CPP was not different among groups. After incubation with L-NAME, CPP increased significantly in all of the groups studied but no differences between the groups were detected, demonstrating that NO has a major role in the maintenance of tonus in the arterial coronary bed regardless of the presence of estrogens and/or ET (Table 2). However, OVX caused a significant impairment in the vasodilation induced by bradykinin, when compared to sham-operated animals. ET prevented the impairment caused by OVX, and that group was able to maintain the same level of vasodilation as observed in the sham group (Figure 1A). When the L-NAME was perfused in the hearts (Figure 1B), the endothelium-mediated responses were profoundly reduced in all groups. In OVX rats, this response was almost abolished. However, the differences between the groups were maintained.

**Table:**
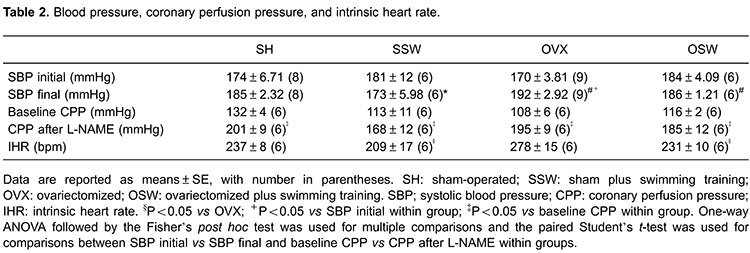

### Superoxide production, antioxidant enzyme expression and activity

**Figure 2:**
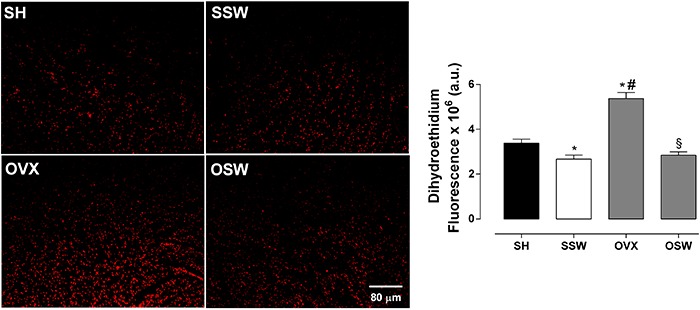
Analysis of superoxide production in cardiac sections by the dihydroethidium fluorescence. SH: sham-operated; SSW: sham plus swimming training; OVX: ovariectomized; OSW: ovariectomized plus swimming training. Data are reported as means±SE for n=4 animals per group. *P<0.05 *vs* SH, ^#^P<0.05 *vs* SSW, and ^§^P<0.05 *vs* OVX (one-way ANOVA followed by Fisher's *post hoc* test).

**Figure 3:**
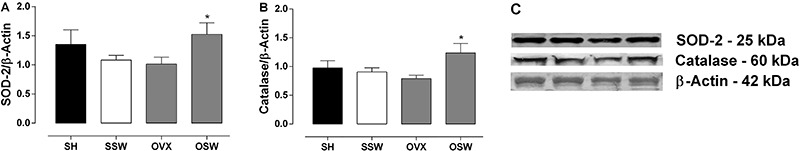
Protein expression of antioxidant enzymes. *A*, Mitochondrial isoform of superoxide dismutase (SOD-2) expression in coronary arteries; *B*, catalase protein expression in coronary arteries, and *C*, representative images of SOD-2, catalase, and β-actin membranes. SW induced an up-regulation in the expression of both enzymes in the OSW group compared to OVX (*P<0.05). SH: sham-operated; SSW: sham plus swimming training; OVX: ovariectomized; OSW: ovariectomized plus swimming training. Data are reported as means±SE (SH: n=6, SSW: n=6, OVX: n=7, OSW: n=7). One-way ANOVA followed by Fisher's *post hoc* test were used for statistical analyses.

**Figure 4:**
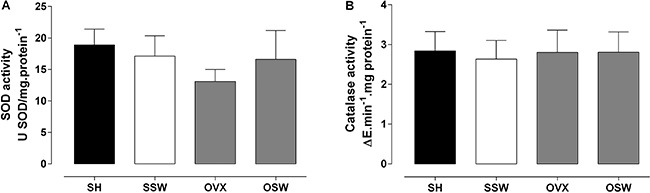
Enzymatic activity of antioxidant enzymes superoxide dismutase (SOD) (*A*) and catalase (*B*) in cardiac tissue. There were no differences among the groups. SH: sham-operated; SSW: sham plus swimming training; OVX: ovariectomized; OSW: ovariectomized plus swimming training. Data are reported as means±SE (SH: n=4, SSW: n=5, OVX: n=5, OSW: n=5). P>0.05 (one-way ANOVA followed by Fisher's *post hoc* test).

Superoxide production, assessed by DHE fluorescence (Figure 2), showed that OVX animals had an augmented oxidative stress compared with the sham groups (P<0.05). However, ET prevented the increase in reactive oxygen species (ROS) production in OVX hypertensive rats (P<0.05 *vs* OVX), demonstrating the antioxidant effects of ET. The expression of antioxidant enzymes SOD-2 and catalase in coronary arteries and the activity of these enzymes in cardiac tissue were verified to determine the possible effect of antioxidant adaptation in the vasodilation promoted by ET. The expression of SOD-2 (Figure 3A), the enzyme that catalyses the dismutation of the superoxide anion (O2^•^) to H_2_O_2_, and the expression of catalase (Figure 3B), which decomposes H_2_O_2_ into water and oxygen, increased significantly in the OSW group compared to the OVX group (P<0.05), suggesting that their expression in coronary arteries are regulated by ET-induced oxidative stress. However, differences among the groups in the cardiac activity of these enzymes were not detected (Figure 4A and B), suggesting that the expression of these enzymes is the major antioxidant adaptation promoted by ET in the absence of estradiol in ovariectomized SHR.

## Discussion

The major results of this study were: 1) OVX impaired endothelium-mediated vasodilation in hypertensive rats, which may be associated with an increase in ROS production; 2) engaging in chronic SW can prevent the endothelial dysfunction that is promoted by the OVX, and 3) the prevention of endothelial dysfunction and oxidative stress observed in hypertensive OVX rats could be mediated by antioxidant effects through increases in coronary expression of SOD-2 and catalase, which could lead to a reduction in oxidative stress, but increases were not observed in the cardiac activities of these enzymes.

Hypertension is an important cardiovascular risk factor, which can lead to significant impairment in the regulation of coronary circulation. Furthermore, menopause can increase the incidence of CVD because of a reduction in the 17β-estradiol concentrations (1). This reduction can also worsen the degree of hypertension in SHR (7,8,16), as also demon strated in our study. Hormone therapy is a treatment commonly used to relieve symptoms associated with menopause and also to prevent CVD. Nevertheless, although studies conducted in animal models have demonstrated the beneficial effects of this therapy on the cardiovascular system (6,23), large clinical trials did not demonstrate such effects (30,31). Thus, the need for other treatment options is of great importance for women during this period due to the high morbidity associated with CHD.

Accordingly, the practice of ET may be an alternative to hormone therapy as many cardiovascular and metabolic benefits have been reported to be associated with regular ET. In this study, although no reduction was observed, SW prevented the worsening of hypertension in both sham-operated and ovariectomized SHR. These results are in agreement with the study of Coimbra et al. (15), which could not detect changes in BP and structural changes in the arteries and heart of hypertensive and ovariectomized animals that trained on a motorized treadmill. Another study (16) demonstrated a hypotensive effect of ET, using a 13-week treadmill training protocol, which was associated with prevention of remodeling in the heart and in the aorta wall.

However, the absence of a decrease in BP and the maintenance of basal coronary perfusion pressure did not significantly alter the occurrence of vascular adaptations to ET. Supporting our hypothesis, SW prevented the impairment of endothelium-dependent vasodilation induced by OVX in SHR rats, with vasodilation remaining at similar levels to those found in the SH group.

In normotensive Wistar rats, 8 weeks of ET not only prevented the coronary endothelial dysfunction of OVX rats, but it also produced a greater response compared to sham-operated animals and those submitted to estrogen therapy (23). Other studies have shown that ET can actually prevent endothelial dysfunction. In male SHR, Roque et al. (32) found that ET on a treadmill can improve endothelial function in isolated coronary arteries and small mesenteric arteries associated with a decrease in oxidative stress and arterial stiffening. In humans, the regular practice of aerobic exercises increases endothelium-derived relaxation stimulated by acetylcholine in both normotensive and hypertensive patients by increasing the release of NO (20). Therefore, according to these results, it can be stated that ET, regardless of the modality practiced and the existence of cardiovascular disease, is a potent stimulus for the improvement and/or maintenance of endothelial function.

To assess the role of NO on the vasodilatory responses stimulated by bradykinin, we inhibited the enzyme nitric oxide synthase. A significant reduction in vasodilation in all groups was found and the difference between the OVX group and the other groups was maintained. These results suggest that NO plays a key role in the endothelium-dependent vasodilator response even with the estrogen deficiency and that endothelial dysfunction caused by OVX occurs through other mechanisms than the reduction of NO production, such as an increase in oxidative stress, which could impair the vasodilation mediated by NO (33,34), prostacyclin (35) and endothelium-derived hyperpolarizing factor (36).

The adaptations induced by ET in relation to antioxidant enzymes in OVX hypertensive rats in our study seem to be primarily mediated by the increase in the expression of the enzymes rather than in their activity. Therefore, this seems to be a major mechanism responsible for the reduction of cardiovascular oxidative stress induced by a long-term ET in this model of postmenopausal hypertension. Consistent with these data, in a previous study from our laboratory with normotensive ovariectomized rats, we observed an increase in the expression of antioxidant enzymes SOD-1 and catalase in the coronary arteries (23). These results are of major importance for improving endothelial function because the model used in this study displays two components that are widely associated with oxidative stress, such as the estrogen deficiency and hypertension.

In conclusion, SW prevents endothelial dysfunction in coronary arteries observed in a model of postmenopausal hypertension. The increase in the expression of antioxidant enzymes seems to be the main factor related to the vascular adaptations promoted by the practice of SW, reducing the oxidative stress and augmenting the bioavailability of NO. Thus, the practice of ET can prevent endothelial dysfunction, and consequently, reduce the risk of CHD in hypertensive postmenopausal women.
